# Role of Feedback during Evaluation in Improving Emergency Medicine Residents’ Skills; an Experimental Study

**Published:** 2017-01-10

**Authors:** Ali Vafaei, Kamran Heidari, Mohammad-Ali Hosseini, Mostafa Alavi-Moghaddam

**Affiliations:** 1 Department of Emergency Medicine, Loghman Hakim Hospital, Shahid Beheshti University of Medical Sciences, Tehran, Iran.; 2 Rehabilitation Management Department, University of Social Welfare and Rehabilitation Sciences, Tehran, Iran.; 3 Department of Emergency Medicine, Imam Hossein Hospital, Shahid Beheshti University of Medical Sciences, Tehran, Iran

**Keywords:** Education, medical, graduate, emergency medicine, learning, formative feedback

## Abstract

**Introduction:**

Evaluation of students’ learning in clinical education system is one of the most important and challenging issues that facilities in this field have been facing. The present study aimed to evaluate the role of feedback during evaluation in increasing emergency medicine residents’ clinical skills.

**Method:**

The present experimental study was performed on all second year emergency medicine residents of two educational hospitals, Tehran, Iran, with switching replications design and before-after method. They were randomly allocated to two groups (with or without feedback) and evaluated three times regarding chest ultrasonography for trauma patients, using direct observation of procedural skills (DOPS) and valid and reliable checklist. Data were analyzed using SPSS 20.

**Results:**

30 emergency medicine residents with the mean age of 36.63 ± 30.30 years were allocated to two equal groups (56.7% male). Studied groups were similar regarding the baseline characteristics. In both groups, obtained scores showed a significant increase from the first to the third evaluation (p < 0.001). Mean scores of first and second evaluations were 10.24 ± 0.77, 17.73 ± 0.46 in feedback receivers and 9.73 ± 0.77 and 12.13 ± 0.47 in others (p < 0.001). Mean third score after switching groups were 18.53 ± 0.22 in feedback receivers and 18.99 ± 0.22 in others (p = 0.213).

**Conclusion:**

Based on the findings of the present study, giving feedback after evaluating the second year emergency medicine residents regarding chest ultrasonography for trauma patients, led to a significant improvement in their scores in future evaluations and consequently their skill.

## Introduction

Training of clinical skills is one of the essential parts of medical education. Medical education facilities have been developed for creating measurable changes in clinical practice (1). Evaluation of students’ learning in clinical education system is one of the most important and challenging issues that facilities in this field have been facing (2). In most clinical courses, evaluation methods do not have sufficient efficiency for assessment of clinical skills (3-5). Common evaluation methods have failed to assess students’ clinical skills accurately, they are not considered as tools for learning. To solve prominent problems, possible strengths and weaknesses of educational facilities were counted and then positive aspects of the educational facilities were improved and their shortcomings were eradicated (6). Investigators in medical education field assessed some methods of clinical evaluation such as performance observation, 360 degree evaluation, objective structured clinical examination, mini-clinical evaluation exercise (Mini-CEX) and direct observation of procedural skills (DOPS), which have been used for evaluation of medical students and their clinical environments. 

Effective assessment of medical students’ clinical skills can improve their motivation and help professors have a more accurate measurement of their actual skills (7). DOPS is known as a new procedure for evaluation of clinical practice skills of medical residents and students, which provides a suitable feedback tool for medical professors (2). Use of formative feedback has positive effects on professional behavior, attitude and communication for medical students (8).

Emergency department is one of the critical hospital wards in which residents must have clinical skills to be prepared for primary health care procedures, diagnosis and treatment of referred patients. Based on the above-mentioned points, the present study aimed to evaluate the role of feedback during evaluation in increasing emergency medicine residents’ clinical skills.

## Methods

***Study design and setting***

The present experimental study was performed on all second year emergency medicine residents of two educational hospitals affiliated with Shahid Beheshti University of Medical Sciences (Imam Hossein and Loghman hakim), Tehran, Iran, from June to November 2015, with switching replications design and before-after method. The study aimed to evaluate the role of feedback during evaluation (FDE) in increasing residents’ skills regarding chest ultrasonography for trauma patients. All researchers adhered to ethical recommendations of Helsinki protocol and confidentiality of participants’ information during the study period.

***Ethical consideration***

Individuals participated in the study by giving informed consent, in addition, data were analyzed as a pool, and the name of individuals was not mentioned. The researchers promised to share the results with the participants on demand. All the patients that participated in rating the skills of residents were selected based on the indications provided in references and were given sufficient information regarding the method of the study. Any patient that wanted to leave the study or the hospital was excluded from the study. Replacements were selected for the 8 patients that decided to leave the study or the hospital. This study did not interfere with the routine diagnostic and treatment process of the patients or impose additional costs on them.

***Participants***

Study participants were selected from second year emergency medicine residents, who had passed their first year promotion exam, using census sampling. All participants were trained regarding chest ultrasonography for trauma patients during their first year of the residency program. They were randomly divided into 2 groups of Loghman Hakim (group 1) or Imam Hossein Hospital (group 2), using simple randomization method. In each studied hospital, one emergency medicine professor, who was informed regarding the study design and DOPS method, was responsible for evaluation of residents. Both attends, who were responsible for evaluation of the participants, were informed regarding the method of the study and its details in a 4-hour coordinator session.

***Procedure***

After preparing essential tools for doing the procedure, participants were evaluated regarding chest ultrasonography for blunt trauma patients using DOPS method.

 Initially, all the participants (separately in both study hospitals) were evaluated using a checklist designed for this purpose and DOPS method in off-duty hours and their scores were recorded. At this stage, after the evaluation was finished, the residents in Loghman Hakim Hospital were given feedback on their weak and strong points for 5 minutes but those in Imam Hossein Hospital were evaluated traditionally and without feedback.

After 2 months, the evaluation was repeated for residents of both hospitals. However, this time evaluation method was switched between the 2 groups and this time residents of Imam Hossein Hospital were given feedback and those in Loghman Hakim Hospital took were evaluated traditionally and without feedback. Finally, about 2 months later, all the second year residents of both hospitals were evaluated again, for the third time.

Duration of evaluation was 15 minutes in all 3 stages, and when the residents were supposed to receive feedbacks it would be organized in 5 minutes. Effort was made to provide similar environmental conditions such as time, place, ultrasonography device, evaluated patients, etc. for both groups.

***Data gathering***

Data gathering tool was a standard (20-item) checklist prepared according to evidence-based texts regarding the requirements that should be met in this procedure (appendix 1). Items of the checklist were designed to evaluate 3 general parts of the procedure including preparation (4 items), carrying out the procedure (13 items), and post-procedure measures (3 items). Each correct answer was given 1 point and for wrong answers, no point was given. Consequently, the maximum and minimum obtainable scores for each participant were 20 and 0, respectively. A score between 0 and 10 was considered as fail and a score between 10 and 20 was considered as pass. Score above 17 was classified as an excellent score.

Validity of the prepared checklist was approved by 10 emergency medicine professors with more than 5 years of experience from various universities. Reliability of the tools was calculated in a pilot study on 15 people and its correlation coefficient was estimated to be 85% based on Cronbach’s alpha.

**Statistical analysis**

Study variables were analyzed by SPSS software version 20. Mean ± standard deviation or frequency and percentage were used for quantitative and qualitative variables, respectively. Paired sample t-test and chi-square were used for comparing quantitative and qualitative variables between study groups. The Greenhouse-Geisser Correction test was used for assessing the impact of repeatedly performing DOPS. All P-values less than 0.05 were considered as significant. 

## Results

***Baseline characteristics***

30 emergency medicine residents with the mean age of 36.63 ± 30.30 (29 - 47) years participated (56.7% male). They were randomly allocated to either Loghman Hakim (group 1) or Imam Hossein hospitals (group 2). Table 1 shows the baseline characteristics of the participants. The two studied groups were similar regarding the baseline characteristics. 25 (83.4%) residents performed chest ultrasonography more than five times (56.7% more than 10 times) and 25 (83.3%) residents believed that chest ultrasonography for trauma patients had medium to high difficulty level. 

***Outcomes***

**Figure 1:**
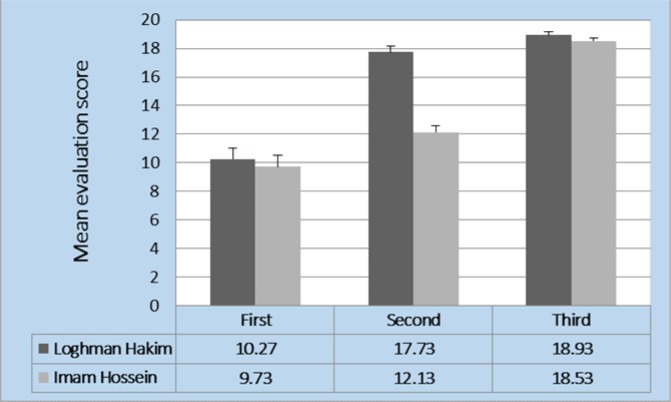
Trend of scores (minimum 0 and maximum 20) in three evaluations of studied groups (p < 0.001, based on Greenhouse-Geisser Correction

Mean scores of the residents in the first, second, and third evaluations were 10.00 ± 0.54, 14.93 ± 0.32, and 18.72 ± 0.15, respectively. Figure 1 shows the scores obtained by the residents for the 3 evaluations done based on the hospital. In both groups, obtained scores showed a significant increase from the first to the third evaluation (p < 0.001) according to Greenhouse-Geisser Correction test.

Mean score obtained for preparation, scan and post-scan items among all residents were 3.34 ± 0.91, 9.48 ± 3.16, and 1.72 ± 0.97, respectively. Tables 2 and 3 compare the scores obtained by each group in the 3 tests qualitatively and quantitatively. Mean scores of first and second evaluations were 10.24 ± 0.77, 17.73 ± 0.46 in feedback receivers and 9.73 ± 0.77 and 12.13 ± 0.47 in others (p < 0.001). Mean third score after switching groups were 18.53 ± 0.22 in feedback receivers and 18.99 ± 0.22 in others (p = 0.213). As can be seen, residents in Loghman Hakim Hospital were significantly better in the second exam both quantitatively and qualitatively.

## Discussion

Based on the findings of the present study, giving feedback after evaluating the second year emergency medicine residents regarding chest ultrasonography for trauma patients, led to a significant improvement in their scores in future evaluations and consequently their skill.

**Appendix 1:** Evaluation checklist of chest ultrasonography for trauma patients using DOPS method

**Items**	**Proper performance**		**Points** ^*^
**Preparation **	**Yes**	**No**	
1. Correctly identifying the patient			
2. Introducing self and being professional			
3. Knowing indications of the procedure			
4. Proper position of patient and device			
**Ultrasonography performance**			
1. Preparing proper tools			
2. Correct selection of probe			
3. Evaluation regarding pneumothorax			
4. Identifying and evaluating the 3 zones			
5. Evaluating all intercostal spaces			
6. Identification of pleural cavity			
7. Lung point sign evaluation			
8. Pleural sliding evaluation			
9. Evaluation of comet tails/B-line			
10. Evaluation of seashore sign in M-mode			
11. Evaluation regarding hemothorax			
12. Right side evaluation of hemothorax			
13. Left side evaluation of hemothorax			
**Post-procedure**			
1. Saving and printing the image			
2. Interpretation of findings			
3. Proper decision based on findings			

* : Each right answer gets 1 point and wrong answers receive no point (minimum 0 and maximum 20).

**Table 1:** Baseline characteristics of participants

**Variable**	**Loghman Hakim**	**Imam Hossein**	**P**
**Mean age (year)**	34.93 ± 5.40	38.33 ± 5.23	0.091
**Sex **			
Male	9 (60)	8 (53.3)	0.71
Female	6 (40)	7 (46.7)	
**Background experience1** **(time)**			
1 -4	3 (20)	2 (13.3)	0.879
5- 9	4 (26.7)	4 (26.7)	
≥ 10	8 (53.3)	9 (60)	
**Background attitude** ^ 2^ ** (difficulty)**			
Low	4 (26.7)	1 (6.7)	0.241
Medium	10 (66.7)	11 (73.3)	
High	1 (6.7)	3 (20)	
**Mean promotion exam score** ^3^	81.06 ± 6.29	82.42 ± 6.27	0.565

Data were presented as mean ± standard deviation or frequency (percentage);

1: Previous experience of chest ultrasonography;

2: Attitude of the resident regarding the difficulty of the procedure;

3: Score in exam taken for promotion from year 1 to year 2 of the residency program (out of 100).

**Table 2:** Comparison of mean evaluation scores between groups

**Evaluation**	**Loghman Hakim**	**Imam Hossein**	**P-value**
**Preparation (0 – 4)**			
First	3.00 ± 0.75	2.1 ± 0.92	0.009
Second	4.00 ± 0.00	3.00 ± 0.85	< 0.0001
Third	3.93 ± 0.26	4.00 ± 0.00	0.326
**Scan (0 – 13)**			
First	6.87 ± 2.39	6.27 ± 2.43	0.501
Second	11.60 ± 0.99	7.33 ± 1.95	< 0.001
Third	12.53 ± 0.74	12.26 ± 0.59	0.287
**Post scan (0 – 3)**			
First	0.40 ± 0.63	1.20 ± 0.67	0.002
Second	2.13 ± 0.35	1.80 ± 0.86	0.176
Third	2.53 ± 0.64	2.27 ± 0.71	0.287
**Total (0 - 20)**			
First	10.26 ± 2.99	9.73 ± 2.94	0.626
Second	17.73 ± 1.09	12.13 ± 2.29	<0.001
Third	18.93 ± 0.96	18.53 ± 0.74	0.213

Data were presented as mean ± standard deviation.

**Table 3:** Comparison of score quality between groups

**Evaluation**	**Loghman Hakim**	**Imam Hossein**	**P-value**
**First **			
Failed	8 (53.3)	5 (33.3)	0.462
Passed	7 (46.7)	10 (66.7)	
Excellent	0 (0)	0 (0)	
**Second **			
Failed	0 (0)	2 (13.3)	< 0.001
Passed	3 (20)	13 (86.7)	
Excellent	12 (80)	0 (0)	
**Third **			
Failed	0 (0)	0 (0)	NA
Passed	15 (100)	15 (100)	
Excellent	0 (0)	0 (0)	

Data were presented as frequency and percentage; NA: not applicable.

Previous studies have shown that assessment of trainees via DOPS can significantly improve skill learning among medical students and residents in some countries (9-11). In most medical education curriculums, DOPS was used for evaluation of main clinical skills such as intravenous cannulation, lumbar puncture, and endotracheal intubation. These procedures were observed in different clinical settings including outpatient clinics and emergency departments. 

Barton et al. and Thomas-Gibson et al. studies indicated that DOPS method causes an increase in residents’ competence in the fields of colonoscopy and endoscopy (12, 13). According to the findings of Hauck S et al., use of DOPS for medical students in ultrasound training field caused a significant increase in their knowledge, confidence, self-motivation and understanding of anatomical structures. 

It is recommended that medical trainers observe medical students while doing the procedure on the patients, and ask them about indications, complications and post procedure cares and immediately give them feedback for future improvement of their skills.

Charleen Liu, who is active in the emergency medicine training field in the United Kingdom, believes that the recent trend in medical education is rapidly moving from traditional routine examinations to collecting evidence of clinical competence and professional behavior observed in clinical environments (work-based learning) through methods such as DOPS, mini-CEX, and case-based discussion (CbD). This is consistent with the highest level of the Miller education pyramid (14).

It is believed that some factors in DOPS such as more practice in implementation of procedure, evaluations in several stages, and giving feedback in each stage can explain improvement of clinical skill among study participants. 

Based on these results of Holmboe et al., DOPS leads to significant changes in students' behavior and competence improvement, as well as increase of teachers’ confidence and satisfaction compared to traditional methods (15, 16).

The results of the current study revealed the significant effect of giving feedback during educational evaluation. Scores of the second evaluation was significantly higher both qualitatively and quantitatively in the group that had received feedback regarding their strong and weak points compared to those who had not. However, after switching the method of evaluation in the second phase, the scores of both groups were at the same level in the third evaluation. Among the secondary outcomes of the study was that it seems that regular evaluation using DOPS itself can improve the skill score of the residents.

## Limitations:

Among the limitations of the present study are the small sample size and evaluation of the residents by 2 separate attends. In addition, considering the previous familiarity of attends with residents we cannot be sure if a conflict of interest existed in their scoring or not. However, in this regard all efforts were made to minimize these limitations by randomly dividing the residents and giving explanations to the evaluators. 

## Conclusion:

Based on the findings of the present study, giving feedback after evaluating the second year emergency medicine residents regarding chest ultrasonography for trauma patients, led to a significant improvement in their scores in future evaluations and consequently their skill.
